# Optimized decision support for selection of transoral robotic surgery or (chemo)radiation therapy based on posttreatment swallowing toxicity

**DOI:** 10.1002/cam4.5253

**Published:** 2022-10-13

**Authors:** Mehdi Hemmati, Carly Barbon, Abdallah S. R. Mohamed, Lisanne V. van Dijk, Amy C. Moreno, Neil D. Gross, Ryan P. Goepfert, Stephen Y. Lai, Katherine A. Hutcheson, Andrew J. Schaefer, Clifton D. Fuller, for the MD Anderson Head and Neck Cancer Symptom Working Group

**Affiliations:** ^1^ Computational Applied Mathematics and Operations Research William Marsh Rice University Houston Texas USA; ^2^ Department of Head and Neck Surgery The University of Texas MD Anderson Cancer Center Houston Texas USA; ^3^ Department of Radiation Oncology The University of Texas MD Anderson Cancer Center Houston Texas USA; ^4^ The University of Texas MD Anderson Cancer Center UTHealth Graduate School of Biomedical Sciences Houston Texas USA; ^5^ Department of Radiation Oncology University Medical Center‐ Groningen Groningen Netherlands

**Keywords:** decision analysis, definitive (chemo)radiation therapy, head‐and‐neck cancer, oropharyngeal squamous cell cancer, posttreatment toxicity, transoral robotic surgery

## Abstract

**Background:**

A primary goal in transoral robotic surgery (TORS) for oropharyngeal squamous cell cancer (OPSCC) survivors is to optimize swallowing function. However, the uncertainty in the outcomes of TORS including postoperative residual positive margin (PM) and extranodal extension (ENE), may necessitate adjuvant therapy, which may cause significant swallowing toxicity to survivors.

**Methods:**

A secondary analysis was performed on a prospective registry data with low‐ to intermediate‐risk human papillomavirus–related OPSCC possibly resectable by TORS. Decision trees were developed to model the uncertainties in TORS compared with definitive radiation therapy (RT) and chemoradiation therapy (CRT). Swallowing toxicities were measured by Dynamic Imaging Grade of Swallowing Toxicity (DIGEST), MD Anderson Dysphagia Inventory (MDADI), and the MD Anderson Symptom Inventory–Head and Neck (MDASI‐HN) instruments. The likelihoods of PM/ENE were varied to determine the thresholds within which each therapy remains optimal.

**Results:**

Compared with RT, TORS resulted in inferior swallowing function for moderate likelihoods of PM/ENE (>60% in short term for all instruments, >75% in long term for DIGEST and MDASI) leaving RT as the optimal treatment. Compared with CRT, TORS remained the optimal therapy based on MDADI and MDASI but showed inferior swallowing outcomes based on DIGEST for moderate‐to‐high likelihoods of PM/ENE (>75% for short‐term and >40% for long‐term outcomes).

**Conclusion:**

In the absence of reliable estimation of postoperative PM/ENE concurrent with significant postoperative PM, the overall toxicity level in OPSCC patients undergoing TORS with adjuvant therapy may become more severe compared with patients receiving nonsurgical treatments thus advocating definitive (C)RT protocols.

## Graphical abstract

The primary aim of transoral robotic surgery (TORS) in overall toxicity level reduction in eligible oropharyngeal squamous cell cancer (OPSCC) patients may be jeopardized in the presence of postoperative extranodal extension (ENE) and/or positive margin (PM) that necessitate adjuvant therapy. Our decision analysis model shows that Moderate‐to‐high likelihoods associated with postoperative PM or ENE that trigger adjuvant therapy leave definitive non‐surgical therapies as the optimal treatment for TORS‐eligible OPSCC patients leading to less overall toxicity burden.

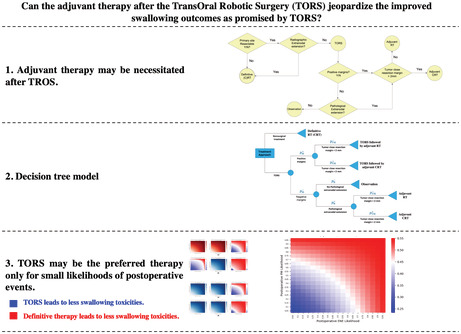

## 1 INTRODUCTION

Recent studies indicate that the incidence of human papillomavirus–associated (HPV+) head‐and‐neck (HNC) cancer has been on a sharp rise, and the incidence of this malignancy is projected to nearly double by the year 2030.^1^, ^2^ With a yearly incidence of 600,000 cases worldwide, there are 62,000 HNC cases annually in the United States with an estimated 13,000 deaths,^3^, ^4^ driven by the endemic rise of oropharyngeal squamous cell cancer (OPSCC).^5^ Historical OPSCC surgical treatment for OPSCC involved surgical procedures including transmandibular and transcervical pharyngotomy, which are associated with significant functional morbidity, notably dysphagia.^6^, ^7^, ^8^ To reduce postoperative morbidity, high‐dose radiation therapy (RT) in combination with chemotherapy became the standard organ‐preserving approach, offering comparable locoregional control and survival. However, nonsurgical chemoradiation therapy (CRT) treatments also put the patient at risk for multiple posttreatment toxicities, including radiation‐associated dysphagia.

**FIGURE 1:**
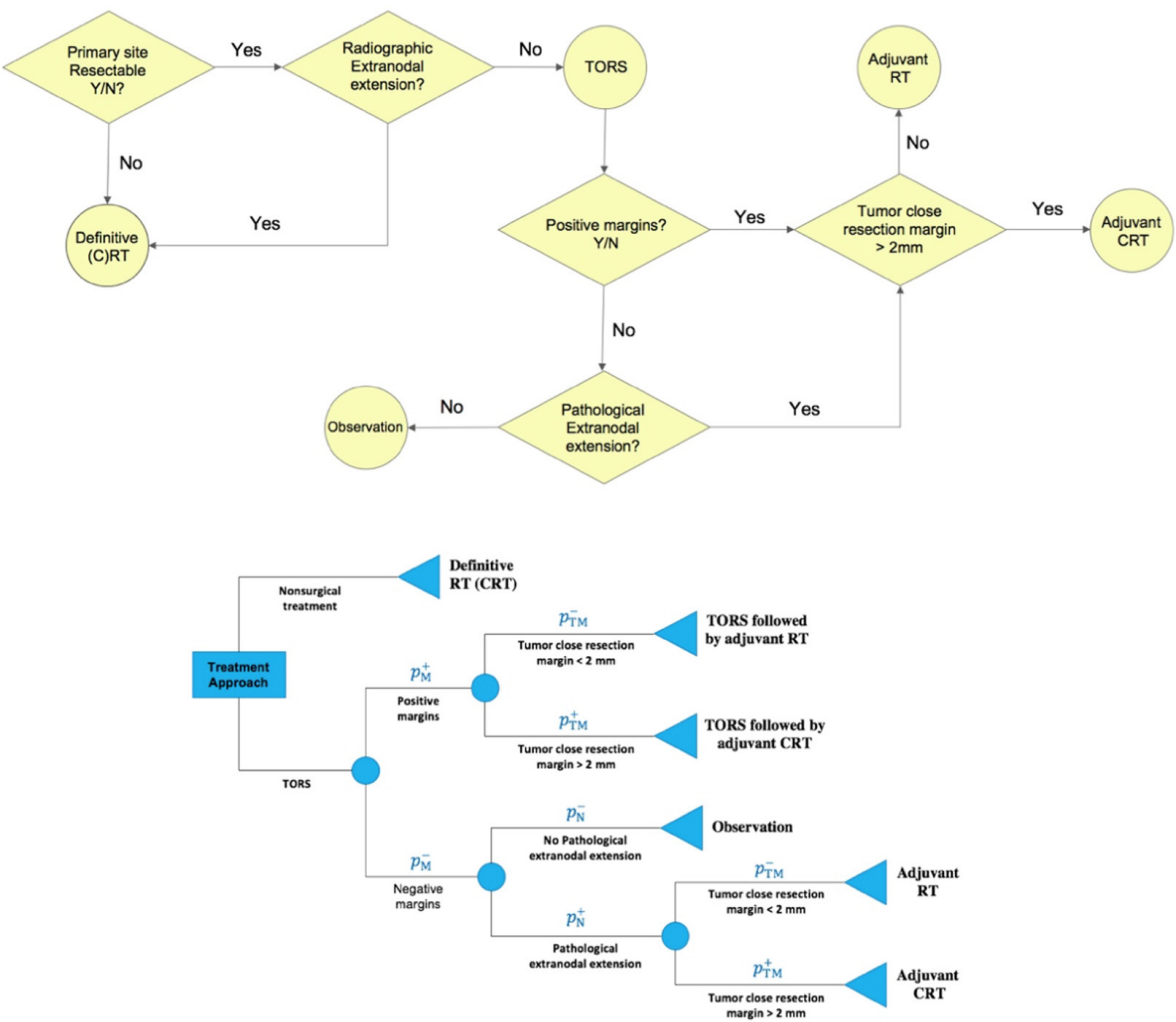
(Top) The underlying process for determining the eligibility of the patient for TORS and its probabilistic outcomes; (Bottom) General decision Tree for TORS decision‐making; scenario 1: definitive RT versus TORS; scenario 2: CRT versus TORS; $p_{M}^{+}\left(p_{M}^{-}\right):$ probability of having (not having) positive margins after surgery; $p_{N}^{+}\left(p_{N}^{-}\right):$ probability of having (not having) extranodal extension after surgery; $p_{\mathrm{TM}}^{+}\left(p_{\mathrm{TM}}^{-}\right):$ probability of having tumor resection margin of more (less) than 2 mm after surgery; CRT, chemoradiation therapy; RT, radiation therapy; TORS, transoral robotic surgery

Transoral robotic surgery (TORS) is a surgical approach that was approved by FDA in 2009 and involves minimal disturbance to critical nerves and swallow musculature of the laryngopharynx, thus promising superior acute postoperative swallowing outcomes compared with traditional open surgical approaches^9^ and therapeutic nonsurgical organ‐preserving regimens in OPSCC^10^ as reported in prospective cohort studies.^11^, ^12^, ^13^, ^14^ Proponents of a primary TORS approach further cite potential for de‐escalation protocols or avoidance of adjuvant therapies, altogether as a major functional advantage of TORS.^10^ Despite TORS promise, it is reported that only 9%–27% of patients treated with frontline TORS avoid postoperative adjuvant RT, and 34%–45% avoid adjuvant CRT.^10^ As probabilistic outcomes of TORS, postoperative positive margins (PMs) or pathological extranodal extensions (ENEs) indicate increased risk of recurrence, and necessitate adjuvant (C)RT (Figure 1 [Top]) with resultant short‐ and long‐term radiation associated toxicities.^14^, ^15^ In a recent study on a cohort of low‐ and intermediate‐risk OPSCC patients receiving definitive (C)RT and TORS (possibly followed by adjuvant therapy), swallowing outcomes (at 3–6 months posttreatment) were reported to be similar regardless of the primary treatment modality.^14^ This suggests that patients undergoing TORS, when followed by adjuvant therapy, may not incur less severe dysphagia compared with receiving definitive CRT as the primary treatment modality.

At present, the relative selection criteria for surgery are primarily qualitative and based on subjective assessment by the physician. Studies suggest that, at least in current practices, physicians are quite poor at predicting the necessity of adjuvant therapy based on presurgical or imaging risk features. This leaves the provider and patient with an upfront pretherapy choice: choose definitive (C)RT with *known* quantized patient‐specific toxicity risk probability *OR* choose an a priori quantifiable toxicity risk of surgery and an *undefined* probability of the risk toxicity of adjuvant therapy. The premise of this study is to define the proportional likelihood of surgical risk features (and resultant indication for adjuvant therapy‐associated toxicity) and determine mathematically optimal decision between primary therapies: (chemo)RT or TORS. Put simply, we address the question of how *numerically confident* the surgeon and radiation oncologist must be in the risk of *pretreatment* pathologic margin positivity or ENE be *to rationally* select TORS for the purposes of minimizing toxicity assuming equivalent locoregional control.

The primary focus of the present study was to develop a decision support tool that aids in selecting the best primary treatment protocol by incorporating the likelihood of postoperative PM and/or ENE to quantify both overall therapy‐related burden level and swallowing function impairment based on short‐ and long‐term toxicities, using an existing prospective data set. The aims of this study were as follows: (i) to quantify the swallowing‐related toxicity levels of definitive therapies and TORS based on subjective and objective instruments using short‐term and long‐term assessments of toxicities and (ii) to determine the required confidence level of likelihood of ENE/PM to determine the optimal primary therapy and its' associated risk level. To achieve these aims, we incorporate the quantified expected swallowing‐related toxicity burden of each primary treatment based on probabilistic postoperative PM and/or ENE events. This is the first application of decision analysis, a widely established tool for decision‐making in uncertain environments. We use this tool to quantify the risk of postoperative swallowing‐related toxicities and the impact on quality of life, measured using highly reliable functional endpoints frequently used in OPSCC.

## 2 METHODS

### 2.1 Study design

This secondary analysis was conducted using prospective registry data from the MD Anderson Oropharynx Cancer Registry (PA14‐0947) Patient‐Reported Outcomes and Function (PROF) Core. The PROF registry enrolls all consenting OPSCC/HNC patients at the University of Texas MD Anderson Cancer Center (MDACC). The sample for this secondary analysis included patients enrolled on PA14‐0914 from March 2015 to February 2018 with the following eligibility criteria: (i) cancer of the oropharynx and (ii) TORS, RT, or CRT as primary treatment approaches at MDACC. All primary treatment was determined by Multidisciplinary Tumor Board. Data analysis occurred under approval of the Institutional Review Board (protocol PA11‐0809).^14^

### 2.2 Demographics

Demographics and treatment characteristics of the cohort are listed in Table 1.^14^

**TABLE 1:** Characteristics of the 257 patients with low‐ to intermediate‐risk oropharyngeal cancer included in the study^14^

Characteristic	All patients	Primary TORS	Primary (C)RT
Enrollment	(*n* = 257)	No adjuvant therapy, *n* = 38	RT Alone, *n* = 30 CRT, *n* = 152
		With adjuvant RT (TORS+RT), *n* = 22	
		With adjuvant CRT (TORS + CRT), *n* = 15	
Total	257	75	182
Age at primary treatment start, mean (SD), y	59.54 (9.07)	58.70 (9.60)	59.89 (8.84)
Sex			
Female	35 (13.6)	10 (13.3)	25 (13.7)
Male	222 (86.4)	65 (86.7)	157 (86.3)
Primary tumor site			
Tonsil	135 (52.5)	38 (50.7)	97 (53.3)
BOT	116 (45.1)	34 (45.3)	82 (45.0)
GPS	6 (2.3)	3 (4.0)	3 (1.6)

### 2.3 Toxicity instruments

Prospective collection of clinician‐ and patient‐graded outcome measures occurred at routine timepoints. The MD Anderson Dysphagia Inventory (MDADI) is a patient‐administered 20‐item questionnaire that evaluates the impact of dysphagia on quality of life. The MDADI includes one question regarding global function and 19 items that focus on the physical, emotional and functional aspects of swallowing, which are pooled and averaged to obtain a composite score (varying from 20 (poor swallowing‐related quality of life) to 100 (optimal swallowing‐related quality of life)).^16^ The MD Anderson Symptom Inventory–Head and Neck Module (MDASI‐HN) is a validated multi‐symptom inventory of patient‐reported swallowing and chewing difficulties based on scores varying from 0 (symptom not present) to 10 (highest imaginable severity of the symptom) and represents a generalizable pan‐symptom toxicity metric.^17^ Lastly, the Dynamic Imaging Grade of Swallowing Toxicity (DIGEST) is a validated and reliable objective tool that measures the presence and severity of pharyngeal dysphagia. The DIGEST conforms to CTCAE criteria for toxicity reporting with a 4‐point grading scale, 0 (no pharyngeal dysphagia), 1 (mild), 2 (moderate), 3 (severe), to 4 (life‐threatening dysphagia).^14^, ^18^ The study was conducted using multiple instruments to avoid risk/decision calibration predicated only based on a subset of the patient toxicity profile, thus accounting for inter‐therapy differential toxicity.

### 2.4 Measures

A total of *six* measures were developed for this study. Each instrument was assessed for all treatment cohorts (TORS, RT alone, CRT alone, TORS with adjuvant RT (TORS+RT), TORS with adjuvant CRT (TORS+CRT)) pretherapy (baseline), 3–6 months and 18–24 months after primary treatment (Table 2). For each cohort, MDADI‐based and MDASI‐based *absolute short‐term deterioration* in swallowing function ($\Delta_{S}^{\text{MDADI}}$ and $\Delta_{S}^{\text{MDASI}}$) were defined as the reduction in MDADI baseline score and the increment in MDASI baseline score, respectively, within 3–6 months. MDADI‐based and MDASI‐based *absolute long‐term deterioration* in swallowing function ($\Delta_{L}^{\text{MDADI}}$ and $\Delta_{L}^{\text{MDASI}}$) were defined analogously with respect to 18–24 months scores. For each treatment cohort, DIGEST‐based *short‐ and long‐term deteriorations* in swallowing functions ($D^{\text{DIGEST}}$ and $R^{\text{DIGEST}}$) were calculated as the fraction of baseline population whose DIGEST baseline grades evolved into any worse grade within 3–6 and 18–24 months after receiving therapy, respectively (Appendix A [A1‐A3]).

**TABLE 2:** MDADI, MDASI, and DIGEST pre‐ and post‐bootstrapping scores pretherapy (baseline), within 3–6 months, and within 18–24 months posttherapy; for DIGEST, total number of cases (bold) and total number of cases whose baseline grades have evolved into inferior grades

Instrument	CRT	RT	TORS+CRT	TORS+RT	TORS
MDADI					
Baseline (mean)	93.26	87.17	94.04	90.18	88.69
3–6 months	81.14	82.02	82.89	83.80	82.55
18–24 months	86.56	81.98	85.16	88.28	86.05
Absolute short‐term deterioration $\Delta_{S}^{\text{MDADI}}$ (bootstrapped)	12.1^a^ ^,^ ^b^	5.34	11.27^a^ ^,^ ^b^	6.43	6.3
Absolute long‐term deterioration $\Delta_{L}^{\text{MDADI}}$ (bootstrapped)	6.71	5.25	8.81	2.05	2.57
MDASI					
Baseline (mean)	0.47	0.99	0.59	0.32	0.81
3–6 months	1.43	1.37	1.09	0.91	1.20
18–24 months	0.99	1.35	0.62	0.54	0.93
Absolute short‐term deterioration $\Delta_{S}^{\text{MDADI}}$ (bootstrapped)	0.95	0.38	0.51	0.59	0.39
Absolute long‐term deterioration $\Delta_{L}^{\text{MDADI}}$ (bootstrapped)	0.51	0.36	0.05	0.22	0.12
DIGEST					
3–6 months incidence with worsen grade	60 (**120**)	10 (**23**)	4 (**10**)	11 (**16**)	5 (**24**)
18–24 months incidence with worsen grade	23 (**66**)	3 (**11**)	3 (**6**)	7 (**7**)	1 (**14**)
Absolute short‐term deterioration $D^{\text{DIGEST}}$ (bootstrapped)	0.5	0.44	0.4	0.69	0.21
Absolute long‐term deterioration $R^{\text{DIGEST}}$ (bootstrapped)	0.35	0.27	0.5	1.00	0.07

*Note*: $\Delta_{S}^{\text{MDADI}}$, $\Delta_{L}^{\text{MDADI}}$, MDADI‐based absolute short‐ and long‐term deterioration; $\Delta_{S}^{\text{MDASI}}$, $\Delta_{L}^{\text{MDASI}}$, MDASI‐based absolute short‐ and long‐term deterioration; $D^{\text{DIGEST}},R^{\text{DIGEST}}$, DIGEST‐based absolute short‐ and long‐term deterioration in swallowing function.

^a^ Indicates where the measure passes the minimum clinically relevant difference.

^b^ Indicates number of patients with worsen condition out of all patients.

### 2.5 Statistical analysis

This study was based on a published analysis conducted by Hutcheson et al.^14^ using the same patient cohort. Bootstrapping‐based resampling^19^, ^20^ was applied to mitigate the effects of unequal sample sizes across treatment cohorts (*n* = 10,000) as well as reducing the variability among of the constructed measures. For MDADI and MDASI scores, bootstrapping was employed based on the empirical distribution computed from the frequency of observed scores. The values of $\Delta_{S}^{\text{MDADI}}$, $\Delta_{L}^{\text{MDADI}}$, $\Delta_{S}^{\text{MDASI}}$, and $\Delta_{L}^{\text{MDASI}}$ were calculated using bootstrapped data sets for each treatment cohort. For DIGEST grades, bootstrapping was employed based on the assumption that the evolution of baseline grades into 3–6 months and 18–24 months grades follows multinomial distribution based on the reported incidence (Appendix C). Next, the values of $R^{\text{DIGEST}}$ and $D^{\text{DIGEST}}$ were calculated using the bootstrapped data set (Table 2).

### 2.6 Decision tree analysis

The aim of this study is to seek the required confidence level with respect to the likelihoods of postoperative ENE/PM for TORS to become the optimal treatment, that is, to outperform definitive (C)RT's *expected* swallowing‐related toxicity burden. An expected‐value decision tree was constructed following the clinical flow depicted in Figure 1 (Top) allowing the measure‐based comparison of definitive (C)RT with deterministic outcomes and TORS with probabilistic outcomes (Figure 1 [Bottom]). Decision trees are extremely efficient for implementing medical guidelines for scenarios with probabilistic outcomes to determine the optimal decision based on expected values of decisions.^21^

The decision tree model was constructed under two distinct scenarios: (i) TORS versus definitive RT and (ii) TORS versus definitive CRT, based on the assumption that the patients studied under each scenario are eligible for both surgical and definitive therapy with comparable locoregional control and survival. For each scenario, the decision model was analyzed for each measure: using the collected measure values (Table 2), the expected toxicity burden of TORS was calculated as a function of postoperative ENE and PM likelihoods ($p_{N}^{+}$ and $p_{M}^{+}$, respectively, ranging from 0 to 1) based on the assumption that when an adjuvant therapy is required, it is equally likely that the patient will undergo adjuvant RT or adjuvant CRT. (Sensitivity analysis was performed to study the effects of this assumption as reported in Appendix B.)

In each scenario, the optimal choice between TORS and the definitive therapy was made based on the observation that for both short‐ and long‐term swallowing‐related toxicity levels, the treatment protocol having lower expected toxicity burden is more favorable. For each scenario and for each short‐or long‐term measure, the *cut‐off* value for TORS ($c_{S},c_{L}$) was computed as the highest expected toxicity burden of TORS under which TORS remains the optimal treatment.

The results of decision tree analysis were demonstrated as 2D heatmaps, for each measure, revealing the combination of likelihoods of postoperative ENE and PM for which TORS swallowing toxicity burden is lower than the definitive therapy. The heatmaps were also employed to derive individual postoperative ENE and PM likelihoods for which definitive therapy becomes the optimal treatment having lower swallowing toxicity level. Finally, to account for the inherent difficulty in pretherapy estimation of postoperative ENE/PM likelihoods, measure‐based *risk associated with* TORS (r) were developed as the fraction of possible combinations of postoperative ENE and PM likelihoods for which definitive therapy offers lower toxicity burden compared with TORS.

## 3 RESULTS

### 3.1 TORS versus definitive RT (Scenario 1)

**FIGURE 2:**
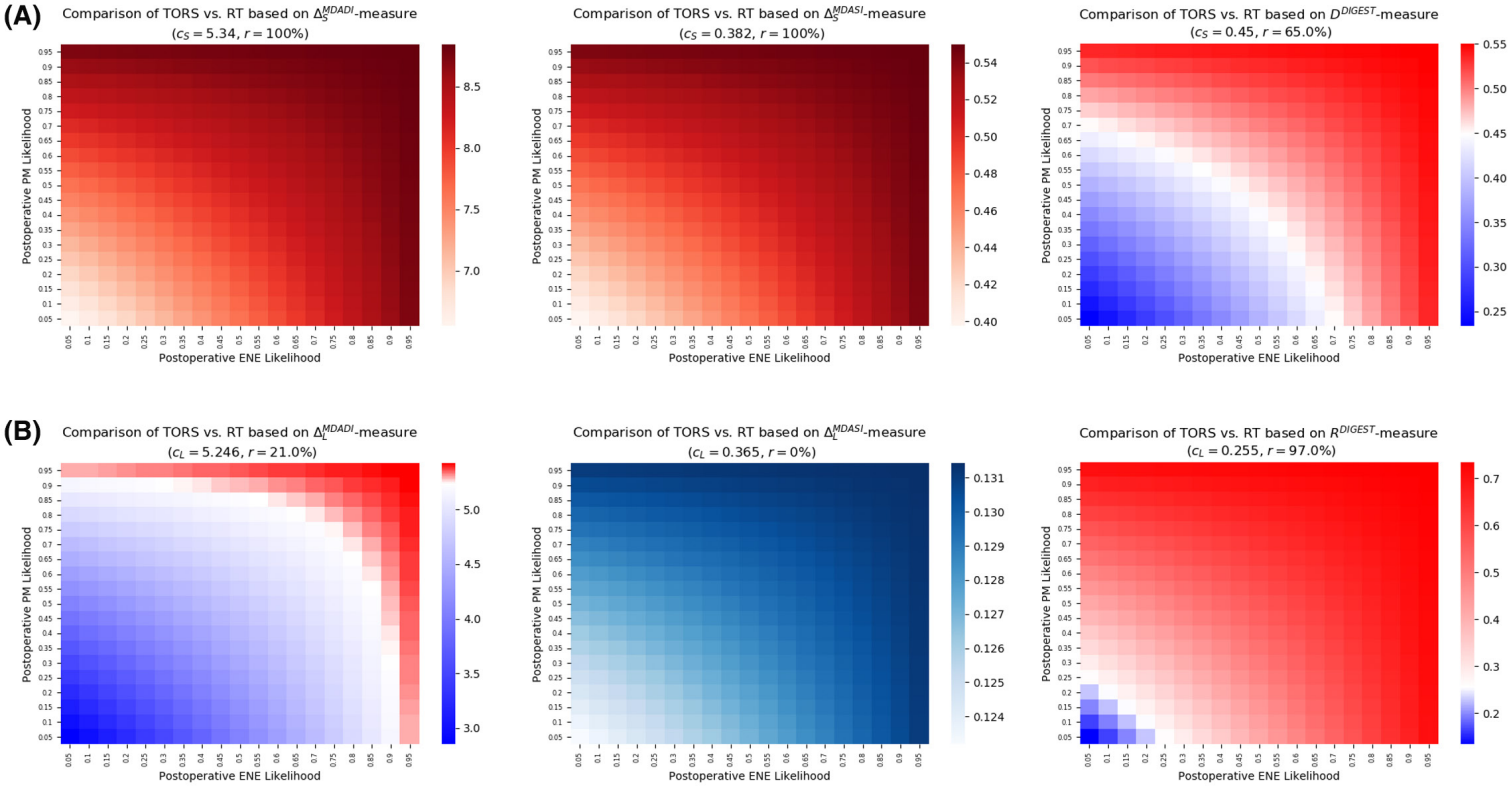
Expected deterioration in swallowing function for the first scenario based on (A) short‐term measures and (B) long‐term measures. $c_{S}$, cut‐off value for TORS; $D^{\text{DIGEST}}$, DIGEST‐based absolute short‐term deterioration in swallowing function; $R^{\text{DIGEST}}$, DIGEST‐based absolute long‐term deterioration in swallowing function; $\Delta_{L}^{\text{MDADI}}$, MDADI‐based absolute long‐term deterioration; $\Delta_{L}^{\text{MDASI}}$, MDASI‐based absolute long‐term deterioration; $\Delta_{S}^{\text{MDADI}}$, MDADI‐based absolute short‐term deterioration; $\Delta_{S}^{\text{MDASI}}$, MDASI‐based absolute short‐term deterioration; r, risk associated with TORS; RT, radiation therapy; TORS, transoral robotic surgery

Figure 2 shows how expected deterioration in swallowing function, computed through six measures (see “Methods” section), can be used to determine the likelihood regions in which either TORS or definitive RT remains the optimal treatment selection. For each of the three short‐term measures, the blue region in Figure 2A indicates the ranges of likelihoods associated with postoperative events (i.e., PM and ENE) for which TORS outperforms definitive RT in terms of swallowing outcomes. The red region, on the other hand, indicate combination of postoperative likelihoods for which TORS results in higher swallowing‐related injuries compared with definitive RT, thus leaving the latter treatment as the optimal choice. Figure 2B provides similar results based on the three long‐term measures.

#### 3.1.1 Short‐term (3–6 month) outcomes

The decision tree analysis using both MDADI and MDASI instruments implied that definitive RT remained the optimal treatment for any postoperative ENE and PM likelihoods (Figure 2A). This is justified based on the observation that the short‐term MDADI‐ and MDASI‐based swallowing‐related toxicities of TORS ($\Delta_{S}^{\text{MDADI}}$ and $\Delta_{S}^{\text{MDASI}}$, respectively) were always more severe compared with definitive RT (Table 2). According to the DIGEST‐measure (presence and severity of dysphagia), if the likelihood associated with either ENE or PM is, at least, 75%, definitive RT remained the optimal treatment. For the cases in which the likelihood of neither ENE or PM is more than 40%, TORS was the optimal treatment. Furthermore, in the absence of pretherapy likelihood estimation of ENE or PM, TORS risk level was at least 65% (according to DIGEST), and definitive therapy outperformed TORS based on MDADI‐ and MDASI‐based measures.

#### 3.1.2 Long‐term (18–24 month) outcomes

For long‐term measures, TORS was the optimal treatment based on MDASI instrument for any confidence level regarding the likelihood of postoperative ENE and PM (Figure 2B). However, based on the MDADI instrument, definitive RT became the optimal treatment when either postoperative ENE or PM are extremely likely (>90%). TORS was the optimal treatment if the likelihood of both postoperative events remained less than 70%. According to the DIGEST instrument, however, definitive RT remained the optimal treatment even if either of the events was likely, at least, 25%. In this case, TORS becomes the optimal treatment only if both events are extremely unlikely (<10%). Finally, in the absence of pretherapy information about ENE or PM likelihood, TORS risk level is at 21% according to MDADI, and 97% according to DIGEST with TORS carrying no risk based on MDASI instrument.

Table 3 summarizes the confidence level of postoperative events likelihoods required to ensure TORS (definitive RT) is the optimal treatment under the first scenario.

**TABLE 3:** Range of likelihoods required for TORS and definitive therapies to become the optimal treatment under the second scenario

Scenario I		
Instrument/measure	Confidence level of postoperative events for which TORS is optimal	Confidence level of postoperative events for which definitive RT is optimal
MDADI		
Short term (3–6 months)	—	Any likelihood associated with ENE and/or PM
Long term (18–24 months)	When both ENE and PM have likelihood <70%	If either of ENE or PM has a likelihood >90%
MDASI		
Short term (3–6 months)	—	Any likelihood associated with ENE and/or PM
Long term (18–24 months)	Any likelihood associated with ENE and/or PM	—
DIGEST		
Short term (3–6 months)	When both ENE and PM have likelihood <40%	If either of ENE or PM has a likelihood >75%
Long term (18–24 months)	When both ENE and PM have likelihood <10%	If either of ENE or PM has a likelihood >25%

Scenario II		
Instrument/measure	Confidence level of postoperative events for which TORS is optimal	Confidence level of postoperative events for which definitive CRT is optimal
MDADI		
Short term (3–6 months)	Any likelihood associated with ENE and/or PM	—
Long term (18–24 months)	Any likelihood associated with ENE and/or PM	—
MDASI		
Short term (3–6 months)	Any likelihood associated with ENE and/or PM	—
Long term (18–24 months)	Any likelihood associated with ENE and/or PM	—
DIGEST		
Short term (3–6 months)	When both ENE and PM have likelihood <55%	If either of ENE or PM has a likelihood >80%
Long term (18–24 months)	When both ENE and PM have likelihood <20%	If either of ENE or PM has a likelihood >40%

Abbreviations: ENE, postoperative extranodal extension; PM, postoperative positive margin.

### 3.2 TORS versus definitive CRT (Scenario 2)

**FIGURE 3:**
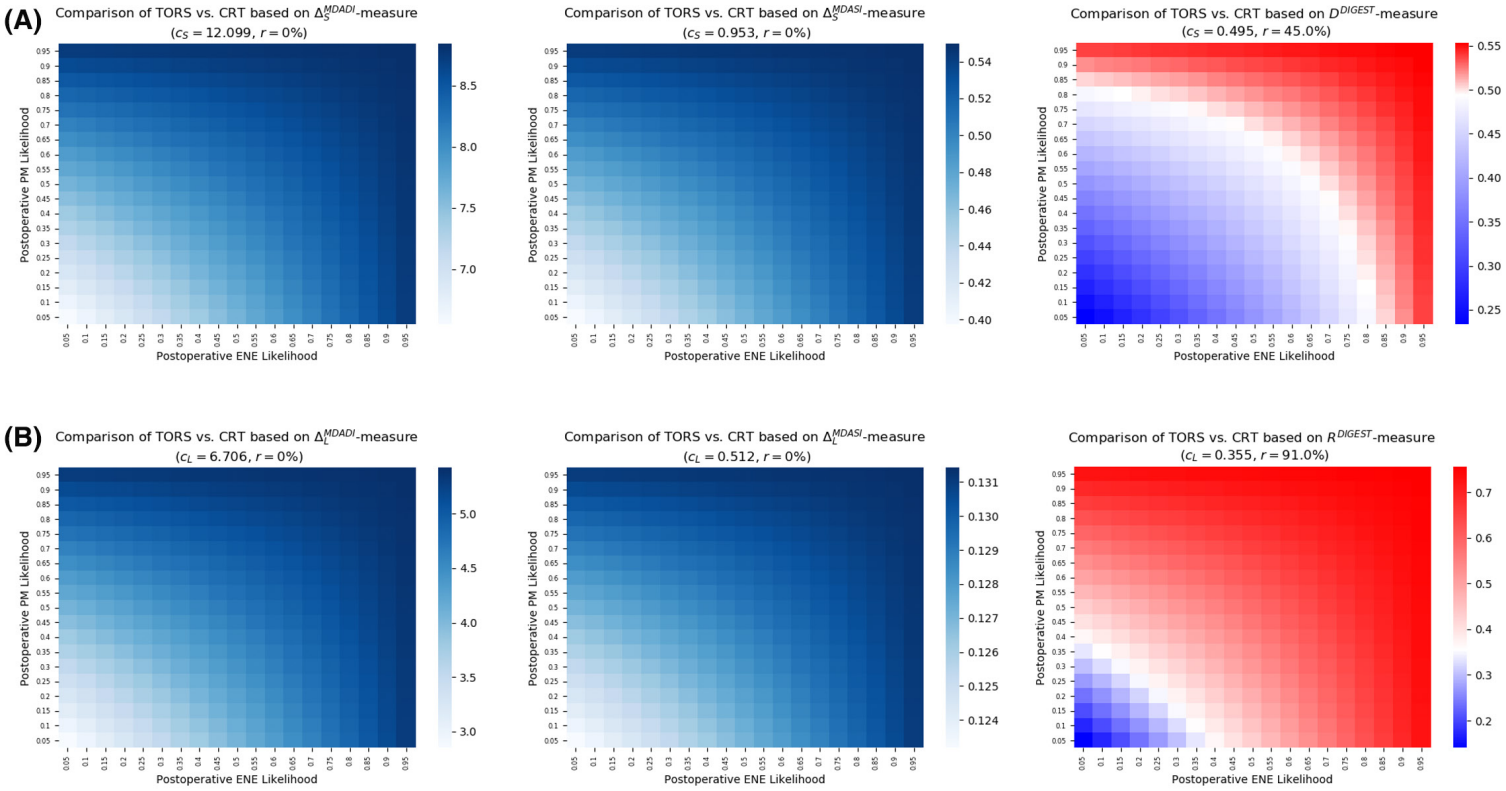
Expected deterioration in swallowing function for the second scenario based on (A) short‐term measures and (B) long‐term measures. $c_{S}$, cut‐off value for TORS; $D^{\text{DIGEST}}$, DIGEST‐based absolute short‐term deterioration in swallowing function; $R^{\text{DIGEST}}$, DIGEST‐based absolute long‐term deterioration in swallowing function; $\Delta_{L}^{\text{MDADI}}$, MDADI‐based absolute long‐term deterioration; $\Delta_{L}^{\text{MDASI}}$, MDASI‐based absolute long‐term deterioration; $\Delta_{S}^{\text{MDADI}}$, MDADI‐based absolute short‐term deterioration; $\Delta_{S}^{\text{MDASI}}$, MDASI‐based absolute short‐term deterioration; CRT, chemoradiation therapy; r, risk associated with TORS; TORS, transoral robotic surgery

Figure 3 demonstrates the comparative analysis for TORS versus definitive CRT using each of the six measures introduced in the “Methods” section. Analogous to Figure 2, for each measure, blue regions in Figure 3 demonstrate the combinations of postoperative events' likelihoods for which TORS is expected to have superior swallowing outcomes compared with definitive CRT. Red regions in Figure 3 indicate the ranges of likelihoods for which definitive CRT is expected to outperform TORS in terms of swallowing injuries. Figure 3A,B provide the results for short‐term and long‐term measures, respectively.

#### 3.2.1 Short‐term (3–6 month) outcomes

When comparing TORS with definitive CRT using short‐term measures, TORS remained the optimal treatment based on both MDADI and MDASI instruments for any likelihoods associated with postoperative ENE and/or PM (Figure 3A). This observation was evident from the related measure values reported in Table 2. Based on the DIGEST instrument, the swallowing toxicity burden of TORS remained higher compared with definitive CRT if the likelihood for any of the postoperative events is more than 80%, thus making definitive CRT the optimal treatment. TORS remained the optimal treatment when both postoperative events have a likelihood of, at most, 55%. Furthermore, in the absence of pretherapy estimation of ENE or PM likelihoods, TORS risk level is at most 45% according to DIGEST, while it carries no risk according to the other instruments.

#### 3.2.2 Long‐term (18–24 month) outcomes

For this scenario, the results of decision tree analysis for long‐term measures were almost similar to those for the first scenario (Figure 3B). TORS remained the optimal treatment based on both MDADI and MDASI instruments being insensitive to the likelihood of postoperative ENE or PM. However, according to the DIGEST instrument, even moderate likelihood (>40%) for either of postoperative events implies the superiority of definitive CRT over TORS. The latter becomes the optimal treatment when both postoperative events have a small likelihood (<20%). Furthermore, when no pretherapy information about the likelihoods is available, TORS carried a risk of level of 91% based on DIGEST, while MDASI and MDASI indicate that there is no risk for TORS (Figure 3B).

A summary of the confidence level of postoperative events likelihoods required for the optimality of TORS (definitive CRT) is given in Table 3.

## 4 DISCUSSION

Since the approval of TORS by FDA as a minimally invasive surgical treatment protocol for HNC patients, there have been several studies reporting on the success of TORS as an option for treatment of early‐stage oropharyngeal carcinomas due to its favorable oncologic outcomes and its potential to mitigate the toxicities incurred by patients in other surgical techniques or primary CRT.^22^, ^23^, ^24^, ^25^ Consequently, TORS has been increasingly used for low‐ to intermediate‐risk OPSCC patients having small‐volume primary tumor and near‐normal baseline function.^14^ However, studies suggest a considerable percentage of patients have undergone postoperative adjuvant CRT,^26^, ^27^, ^28^ despite being theoretically believed to be candidates for surgical therapy alone. The current data suggest that surgeons and radiation oncologists are decidedly poor at predicting whether a patient will require adjuvant treatment from pretherapy exam, and thus many patients offered surgical resection are in fact being offered *not* TORS alone, but *rather some unquantified probability of double‐ or triple‐modality therapy* (and the concomitant additional toxicities therefrom). This is primarily due to absence of extreme *pre*surgical certitude regarding of *post*‐TORS histopathological features, which makes it a challenging decision‐making problem to choose between initial TORS or definitive nonsurgical treatment protocols.

Decision analysis provides an integrated framework to study decision‐making scenarios that involve uncertain outcomes. The decision analysis model developed in this study incorporates the imputed pretherapy physic‐assessed statistical likelihood of the two major postoperative indicator events that trigger adjuvant therapy: *pretherapy* physician‐estimated probability of margin positivity and ENE. Through quantifying *posttherapy* swallowing‐related toxicities using well‐established patient‐reported and objective instruments, the model in this study captures the probabilistic outcomes of TORS which in comparison with the definitive (C)RT's outcome can aid the clinical team in choosing the optimal treatment protocol. While objective instruments are developed based on standard test results, hence providing more concrete results in comparing the change in the patient's quality of life, they might be less indicative of the patient's lived experience. The developed decision support tool in this study is developed under the premise that the physician will include both objective and patient‐reported measures when deciding about the optimal therapy. The results of this model can also be utilized to compute the risk level associated with TORS in developing higher swallowing‐related toxicity burden compared with definitive (C)RT in the absence of pretherapy estimation of the likelihood of postoperative events that can trigger the need for adjuvant therapy.

Three observations are notable from the current analysis: (i) there are distinctly different optimal choices based on the probability of postoperative events that differ whether radiotherapy‐alone or chemoradiotherapy is the comparator for surgical treatment; (ii) there are divergent optimal choice of therapy regarding subjective multisymptom (MDASI), subjective swallowing (MDADI) or objective swallowing (DIGEST) is the toxicity metric of interest; and (iii) the choice of therapy based on early (3–6 month) swallowing outcomes may not reflect the optimal therapy selection for later time‐points (18–24 months).

The decision analysis model in this work has its own limitations. It currently relies on a single institutional database with a cohort of 257 patients. Bootstrapping was used to mitigate the effects of small‐size population allowing the model to make assumptions as “real‐life” as possible. Furthermore, expected‐value decision analysis has its own disadvantages, namely the sensitivity to the probability values as well as measures. Sensitivity analysis was performed to determine the variation of TORS risk level as a function of the likelihood of postoperative events as well as associated quantified short‐ and long‐term toxicity of all treatment protocols. We would like to emphasize that while our model has been constructed based on single‐institutional data, it can be easily instantiated using validated possibly multi‐institutional data or even from randomized trials.

Ultimately, the aim of this effort is to quantize decision‐making for HNC/OPSCC patients eligible for alternative treatment protocols. The vast majority of cases selected for definitive or surgical therapy (potentially followed by adjuvant radiotherapy) are typically made using heuristic physician‐decision processes, which appear to be speciously high estimates of the potential for single‐modality surgery. However, advanced approaches such as improved standardized radiologic assessment,^29^ AI‐assisted imaging analysis, or risk‐models^30^ could improve outcomes by bringing quantitative decision support to surgeons and radiation oncologists. Furthermore, these data serve to define preoperative assessment tools for decision support for future explorations.

## 5 CONCLUSION

Our models demonstrated optimal decision thresholds for selection of surgical possibly with adjuvant therapy or organ preservation with (chemo)radiotherapy based on clinically‐representative subjective and objective toxicity outcomes. The resultant thresholds for physician certainty for prediction of clinical risk features necessitating adjuvant therapy should be considered with these decision tools as a component of multidisciplinary patient‐centric therapy selection for early‐stage oropharyngeal cancer patients.

## AUTHOR CONTRIBUTION

Mehdi Hemmati, Clifton D. Fuller, and Andrew J. Schaefer contributed to the study concept and design. Mehdi Hemmati implemented the decision support tool and performed the analysis. Mehdi Hemmati and Carly Barbon contributed to the data analysis. Abdallah S.R. Mohamed and Lisanne V. van Dijk contributed to data collection and interpretation. Amy C. Moreno, Neil D. Gross, Ryan P. Goepfert, Stephen Y. Lai, and Katherine A. Hutcheson contributed to the analysis and interpretation of the results. Mehdi Hemmati drafted the manuscript. All authors contributed to the interpretation of data and provided feedback on the manuscript.

## CONFLICT OF INTEREST

Dr. Fuller received/receives funding and salary support from directly related to this project from: NIH National Institute of Dental and Craniofacial Research (NIDCR) Academic Industrial Partnership Grant (R01DE028290); NIDCR Establishing Outcome Measures for Clinical Studies of Oral and Craniofacial Diseases and Conditions award (R01DE025248); NIH/NSF NCI Smart Connected Health Program (R01CA257814). Dr. Fuller received/receives funding and salary support from directly unrelated to this project from: NCI Parent Research Project Grant (R01CA258827); NCI Ruth L. Kirschstein NRSA Institutional Research Training Grant (T32CA261856); NIH NIDCR Exploratory/Developmental Research Grant Program (R21DE031082); National Institutes of Health (NIH) National Cancer Institute (NCI) Early Stage Development of Technologies in Biomedical Computing, Informatics, and Big Data Science Program (R01CA214825); NSF/NIH Joint Initiative on Quantitative Approaches to Biomedical Big Data program (R01CA225190); NIH National Institute of Biomedical Imaging and Bioengineering (NIBIB) Research Education Programs for Residents and Clinical Fellows Grant (R25EB025787); NCI Early Phase Clinical Trials in Imaging and Image‐Guided Interventions Program (1R01CA218148); NIH/NCI Cancer Center Support Grant (CCSG) Pilot Research Program Award from the UT MD Anderson CCSG Radiation Oncology and Cancer Imaging Program (P30CA016672); Small Business Innovation Research Grant Program a sub‐award from Oncospace, Inc. (R43CA254559); The Human BioMolecular Atlas Program (HuBMAP) Integration, Visualization & Engagement (HIVE) Initiative (OT2OD026675) sub‐award; Patient‐Centered Outcomes Research Institute (PCS‐1609‐36,195) sub‐award from Princess Margaret Hospital; National Science Foundation (NSF) Division of Civil, Mechanical, and Manufacturing Innovation (CMMI) grant (NSF 1933369). Dr. Fuller receives grant and infrastructure support from MD Anderson Cancer Center via the Charles and Daneen Stiefel Center for Head and Neck Cancer Oropharyngeal Cancer Research Program; the Program in Image‐guided Cancer Therapy; and the NIH/NCI Cancer Center Support Grant (CCSG) Radiation Oncology and Cancer Imaging Program (P30CA016672). Dr. Fuller has received direct industry grant/in‐kind support, honoraria, and travel funding from Elekta AB. TCS was supported by The University of Texas Health Science Center at Houston Center for Clinical and Translational Sciences TL1 Program (TL1 TR003169).

## ETHICS STATEMENT

The ethics approval was obtained from the University of Texas MD Anderson Cancer Center Institutional Review Board with protocol number: PA11‐0809.

## Supporting information


Appendix A

Appendix B

Appendix C

## Data Availability

The data that support the findings of this study are openly available in *figshare* at the following URL: https://doi.org/10.6084/m9.figshare.19636755.v1
