# Supplementary material for: Optimized decision support for selection of transoral robotic surgery or (chemo)radiation therapy based on posttreatment swallowing toxicity
Source: Cancer Med. 2022 Oct 13;12(4):5088–98. doi: 10.1002/cam4.5253 (PMC9972156; doi:10.1002/cam4.5253)
Supplement: Supplementary file 1 — Appendix A [file CAM4-12-5088-s001.docx]

**Appendix A1: Computation of MDADI-based short-term and long-term scores**

Based on the responses from the patients treated via treatment approach X (i.e., TORS, D_RT, D_CRT, TORS+RT, TORS+CRT, as stated in Table 1, the *absolute* short- and long-term deterioration in swallowing function based on MDADI score were computed as

$$\Delta_{S}^{MDADI}(X)=\mathrm{MDADI}_{baseline}\left( X \right)-\mathrm{MDADI}_{3-6}\left( X \right),$$

and

$$\Delta_{L}^{MDADI}\left( X \right)=\mathrm{MDADI}_{baseline}\left( X \right)-\mathrm{MDADI}_{18-24}\left( X \right),$$

respectively, where $\mathrm{MDADI}_{baseline}(X)$, $\mathrm{MDADI}_{3-6}\left( X \right),$and $\mathrm{MDADI}_{12-24}(X)$ are the patient-reported MDADI scores prior to receiving treatment $X$, within 3-6 months, and within 18-24 months after receiving treatment $X$, respectively, for a given treatment $X$.

**Appendix A2: Computation of MDASI-based short-term and long-term scores**

Participating patients completed the MDASI-HN questionnaire before (baseline), within 3-6 month, and 18-24 months after therapy. The MDASI-based *absolute* short- and long-term deterioration in swallowing function for treatment approach X were computed as

$$\Delta_{S}^{MDASI}(X)=\mathrm{MDASI}_{3-6}\left( X \right)-\mathrm{MDASI}_{baseline}\left( X \right),$$

and

$$\Delta_{L}^{MDADI}\left( X \right)=\mathrm{MDADI}_{18-24}\left( X \right)-\mathrm{MDADI}_{baseline}\left( X \right),$$

respectively.

**Appendix A3: Computation of DIGEST-based short-term and long-term grades**

In this study, measures based on pre- and post-therapy DIGEST grades were employed. The *DIGEST-based long-term deterioration* in the swallowing function due to treatment X was defined as

$$R^{DIGEST}\left( X \right)=\frac{n_{L}\left( X \right)}{N_{L}(X)},$$

where $n_{L}\left( X \right)$ is the number of patients whose DIGEST grade increased within 18-24 months compared to their baseline grades. Also, $N_{L}\left( X \right)$ is the number of patients for whom DIGEST grade was computed within 18-24 months after the therapy. Furthermore, the *DIGEST-based short-term deterioriation* in the swallowing function due to treatment $X$ was defined as

$$D^{DIGEST}\left( X \right)=\frac{n_{S}\left( X \right)}{N_{S}(X)},$$

with $n_{S}\left( X \right)$ being the number of patients whose DIGEST grade increased within 3-6 months compared to their baseline grades. $N_{S}\left( X \right)$ is defined analogously to $N_{L}\left( X \right)$ for the period of 3-6 months after treatment $X$.
