# Supplementary material for: Optimized decision support for selection of transoral robotic surgery or (chemo)radiation therapy based on posttreatment swallowing toxicity
Source: Cancer Med. 2022 Oct 13;12(4):5088–98. doi: 10.1002/cam4.5253 (PMC9972156; doi:10.1002/cam4.5253)
Supplement: Supplementary file 2 — Appendix B [file CAM4-12-5088-s003.docx]

**Appendix B: Sensitivity analysis for the likelihood of having significant postoperative tumor resection margin**

The heatmaps were reproduced for extremely low and high values of the probability of having significant postoperative tumor resection margin ($p_{TM}^{+}$) in order to study the effect on the TORS risk level compared to definitive therapies.

1. **TORS vs. definitive RT**
   1. **Short-term outcomes (**$\boldsymbol{p}_{\boldsymbol{TM}}^{\boldsymbol{+}}\boldsymbol{=0.1}$**)**

**
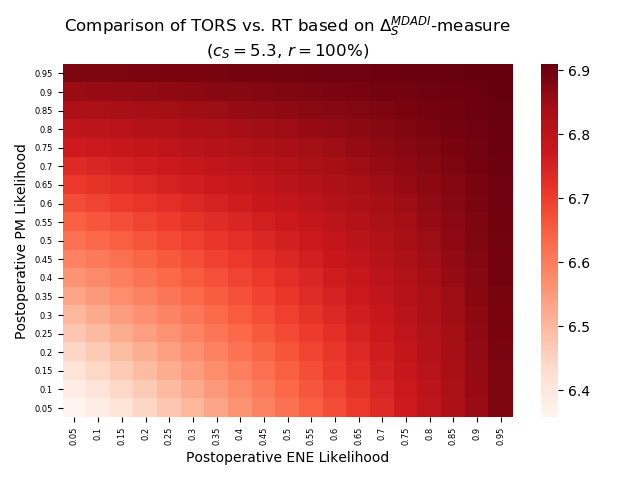

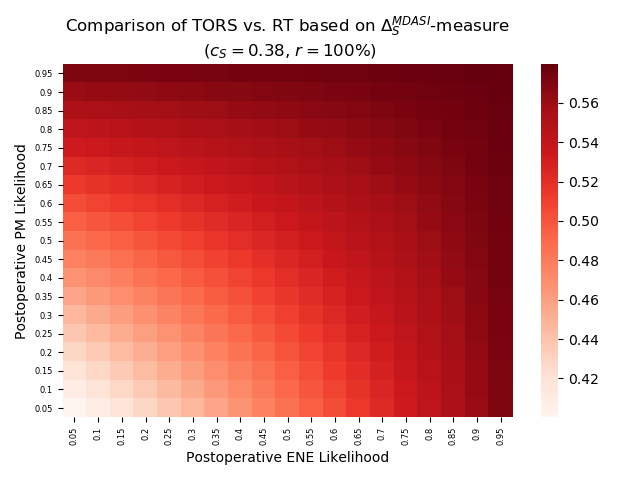

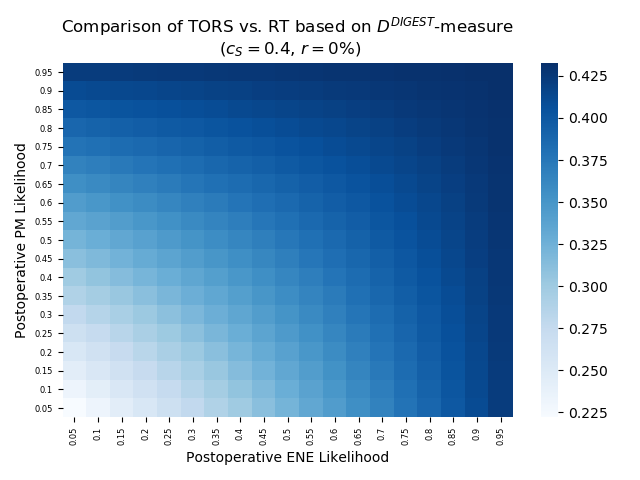
**

Figure B1: Expected deterioration in swallowing function due to TORS and definitive RT based on short-term measures (left) MDADI, (center) MDASI, (right) DIGEST. $\Delta_{S}^{MDADI}$: MDADI-based absolute short-term deterioration; $\Delta_{S}^{MDASI}$: MDASI-based absolute short-term deterioration; $D^{DIGEST}$: DIGEST-based absolute short-term deterioration in swallowing function; $c_{S}$: cut-off value for TORS; and $r:$ risk associated with TORS for $p_{TM}^{+}=0.1.$

- 1. **Short-term outcomes (**$\boldsymbol{p}_{\boldsymbol{TM}}^{\mathbf{+}}\mathbf{=0.9}$**)**

**
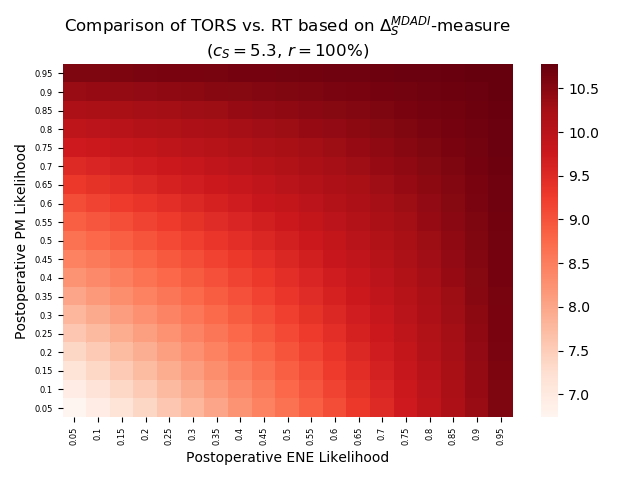

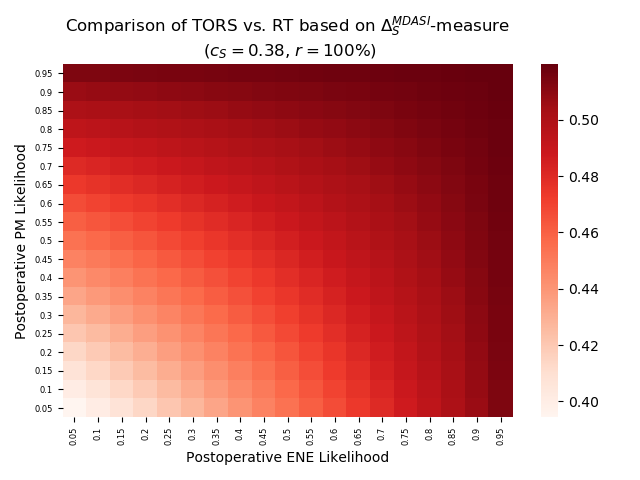

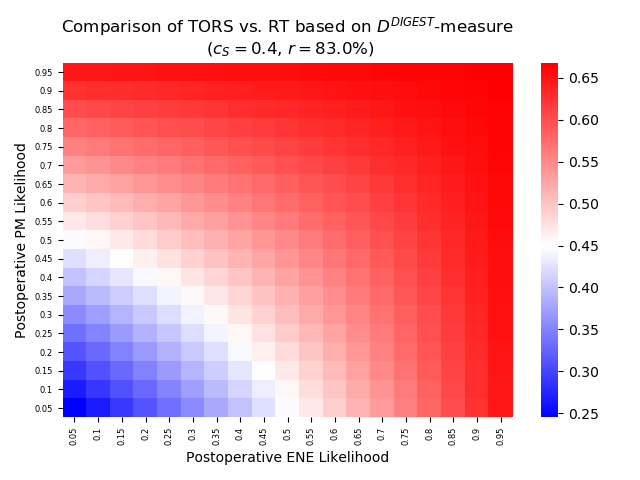
**

Figure B2: Expected deterioration in swallowing function due to TORS and definitive RT based on short-term measures (left) MDADI, (center) MDASI, (right) DIGEST. $\Delta_{S}^{MDADI}$: MDADI-based absolute short-term deterioration; $\Delta_{S}^{MDASI}$: MDASI-based absolute short-term deterioration; $D^{DIGEST}$: DIGEST-based absolute short-term deterioration in swallowing function; $c_{S}$: cut-off value for TORS; and $r:$ risk associated with TORS for $p_{TM}^{+}=0.9.$

- 1. **Long-term outcomes (**$\boldsymbol{p}_{\boldsymbol{TM}}^{\mathbf{+}}\mathbf{=0.1}$**)**


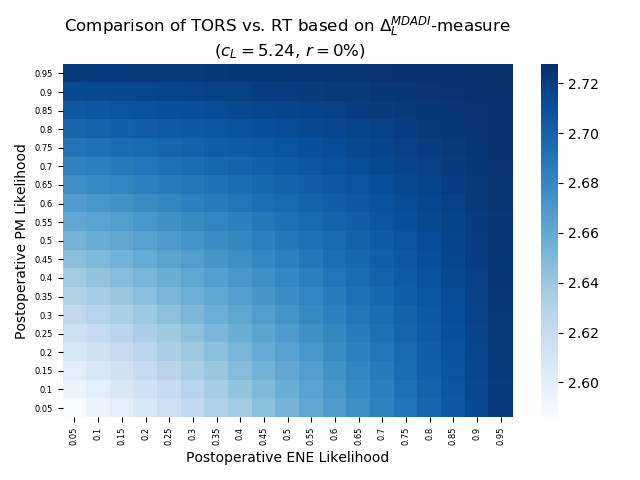

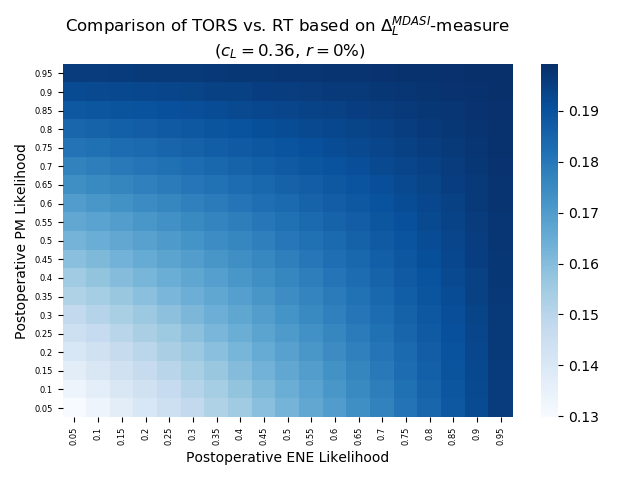
**
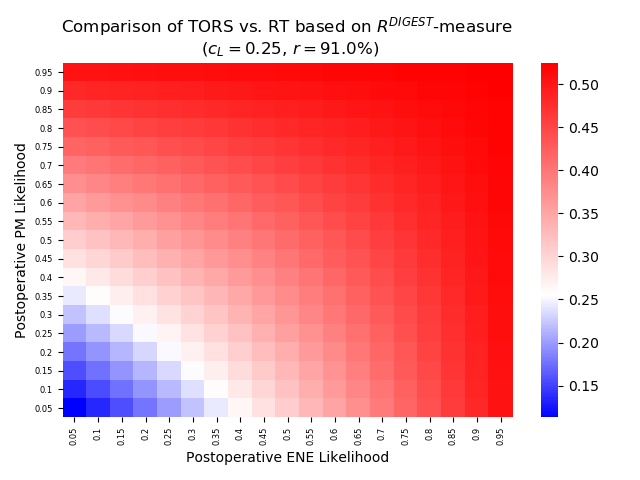
**

Figure B3: Expected deterioration in swallowing function due to TORS and definitive RT based on long-term measures (left) MDADI, (center) MDASI, (right) DIGEST. $\Delta_{S}^{MDADI}$: MDADI-based absolute short-term deterioration; $\Delta_{S}^{MDASI}$: MDASI-based absolute short-term deterioration; $D^{DIGEST}$: DIGEST-based absolute short-term deterioration in swallowing function; $c_{S}$: cut-off value for TORS; and $r:$ risk associated with TORS for $p_{TM}^{+}=0.1.$

- 1. **Long-term outcomes (**$\boldsymbol{p}_{\boldsymbol{TM}}^{\mathbf{+}}\mathbf{=0.9}$**)**

**
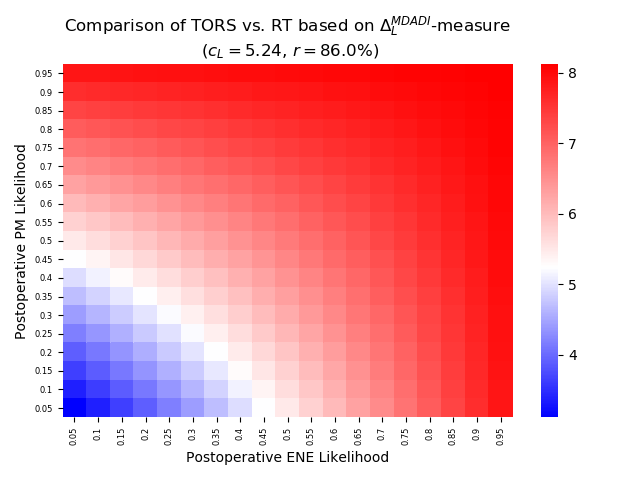

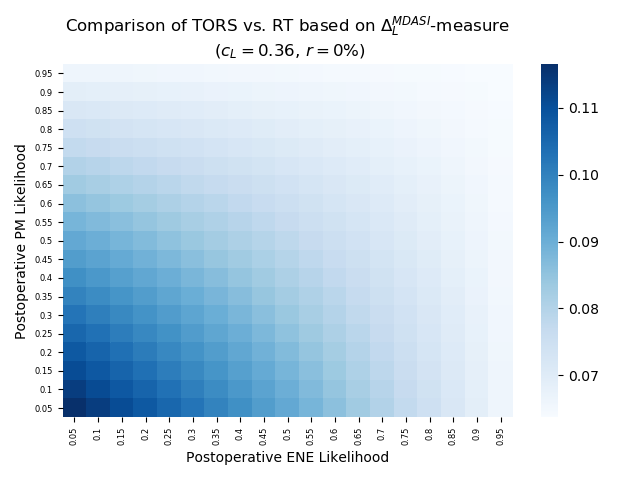
***
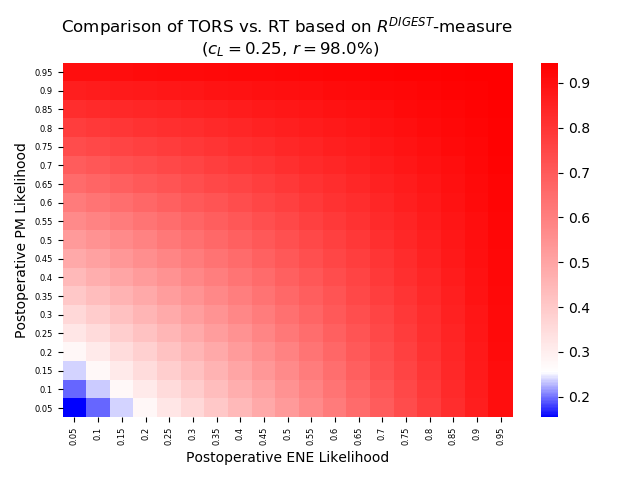
*

Figure B4: Expected deterioration in swallowing function due to TORS and definitive RT based on short-term measures (left) MDADI, (center) MDASI, (right) DIGEST. $\Delta_{S}^{MDADI}$: MDADI-based absolute short-term deterioration; $\Delta_{S}^{MDASI}$: MDASI-based absolute short-term deterioration; $D^{DIGEST}$: DIGEST-based absolute short-term deterioration in swallowing function; $c_{S}$: cut-off value for TORS; and $r:$ risk associated with TORS for $p_{TM}^{+}=0.9.$

Table B1 summarizes the risk level variation associated with TORS (when compared to definitive RT), as a function of tumor resection margin, in the absence of pre-therapy information about the postoperative ENE and PM likelihoods.

Table B1: Sensitivity of risk level associated with TORS (vs. definitive RT) as a function of postoperative tumor resection margin. $p_{TM}^{+}$: probability of having tumor resection margin > 2mm; $r$: TORS risk level; (I): Insensitive to $p_{TM}^{+}$; (HS): Highly sensitive to $p_{TM}^{+}$;; (LS): Low sensitive to $p_{TM}^{+}$.

|  | $\boldsymbol{p}_{\boldsymbol{TM}}^{\mathbf{+}}$ **= 50%** | **Risk sensitivity to** $\boldsymbol{p}_{\boldsymbol{TM}}^{\mathbf{+}}$ | |  |
| --- | --- | --- | --- | --- |
| **Short-term toxicity level** | $65\%\leq r\leq100\%$  TORS carries high risk  (conclusive across all instruments) | MDADI | $r=100\%$ (I) | |
|  |  | MDASI | $r=100\%$ (I) | |
|  |  | DIGEST | $r\leq83\%$ (HS) | |
| **Long-term toxicity level** | $0\%\leq r\leq97\%$  TORS risk varies based on the instrument  (inconclusive across all instruments) | MDADI | $r\leq86\%$ (HS) | |
|  |  | MDASI | $r=0\%$ (I) | |
|  |  | DIGEST | $91\%\leq r\leq98\%$ (I) | |

1. **TORS vs. definitive CRT**
   1. **Short-term outcomes (**$\boldsymbol{p}_{\boldsymbol{TM}}^{\mathbf{+}}\mathbf{=0.1}$**)**

**
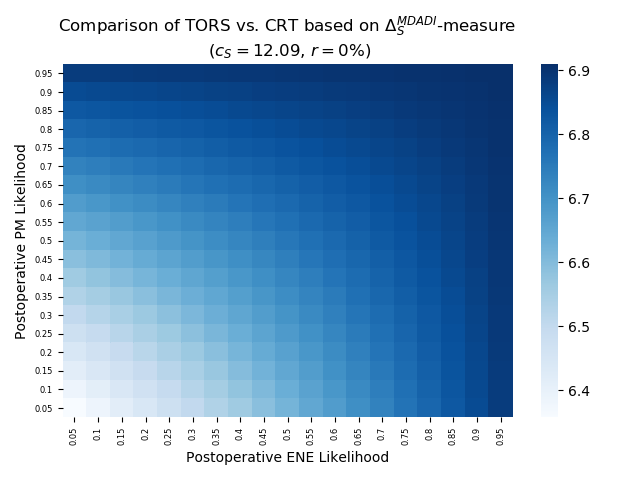

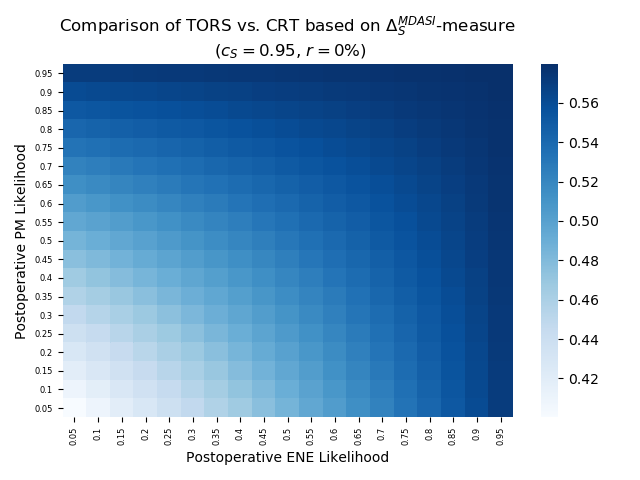

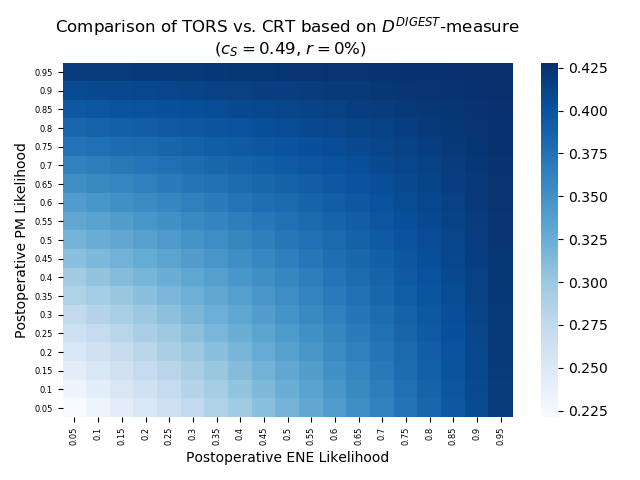
**

Figure B5: Expected deterioration in swallowing function due to TORS and definitive CRT based on short-term measures (left) MDADI, (center) MDASI, (right) DIGEST. $\Delta_{S}^{MDADI}$: MDADI-based absolute short-term deterioration; $\Delta_{S}^{MDASI}$: MDASI-based absolute short-term deterioration; $D^{DIGEST}$: DIGEST-based absolute short-term deterioration in swallowing function; $c_{S}$: cut-off value for TORS; and $r:$ risk associated with TORS for $p_{TM}^{+}=0.1.$

- 1. **Short-term outcomes (**$\boldsymbol{p}_{\boldsymbol{TM}}^{\mathbf{+}}\mathbf{=0.9}$**)**

**
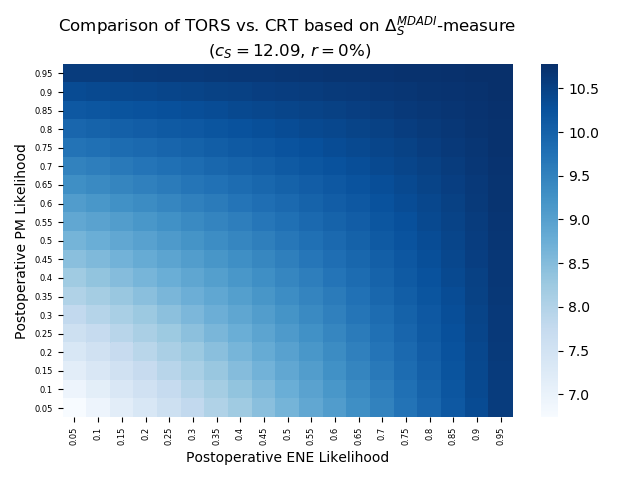

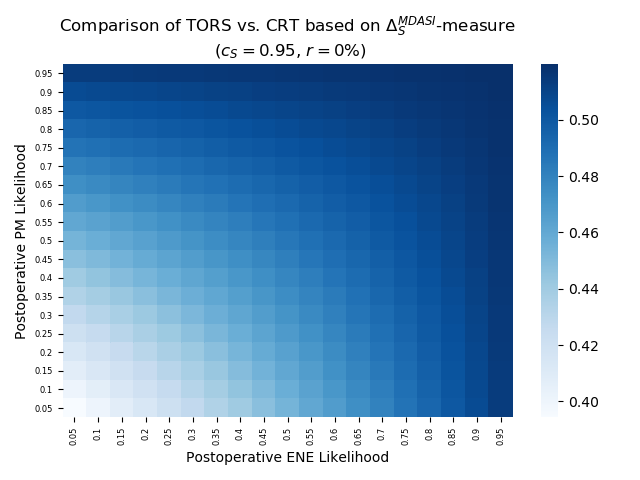

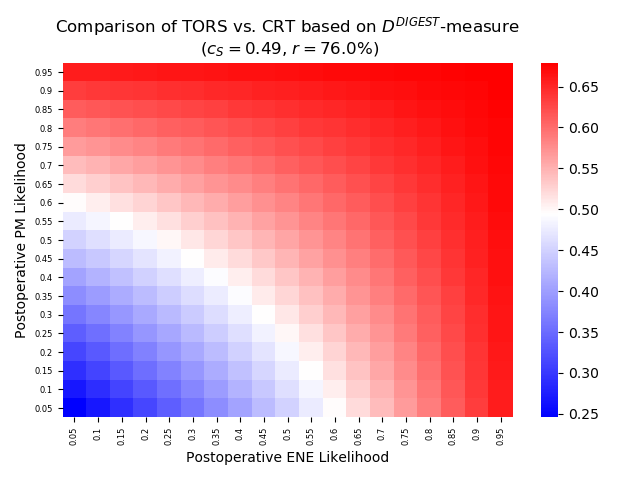
**

Figure B6: Expected deterioration in swallowing function due to TORS and definitive CRT based on short-term measures (left) MDADI, (center) MDASI, (right) DIGEST. $\Delta_{S}^{MDADI}$: MDADI-based absolute short-term deterioration; $\Delta_{S}^{MDASI}$: MDASI-based absolute short-term deterioration; $D^{DIGEST}$: DIGEST-based absolute short-term deterioration in swallowing function; $c_{S}$: cut-off value for TORS; and $r:$ risk associated with TORS for $p_{TM}^{+}=0.9.$

- 1. **Long-term outcomes (**$\boldsymbol{p}_{\boldsymbol{TM}}^{\mathbf{+}}\mathbf{=0.1}$**)**

**
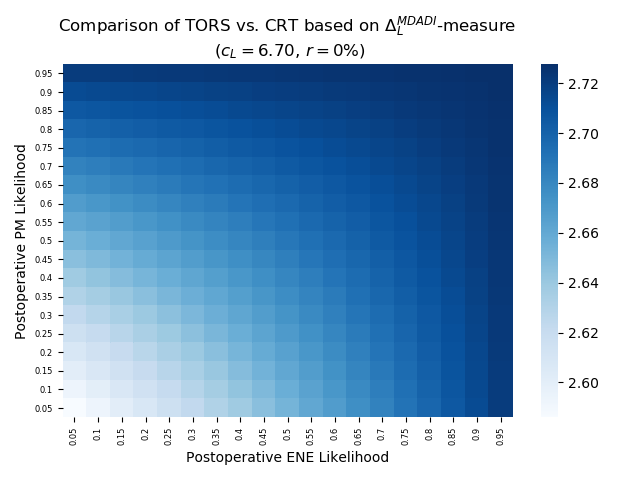

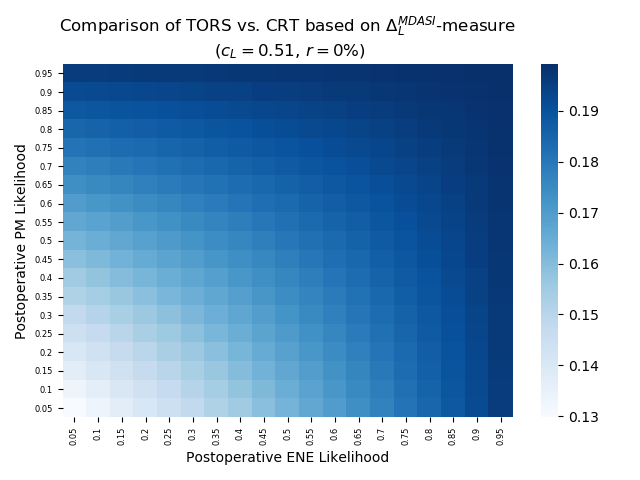

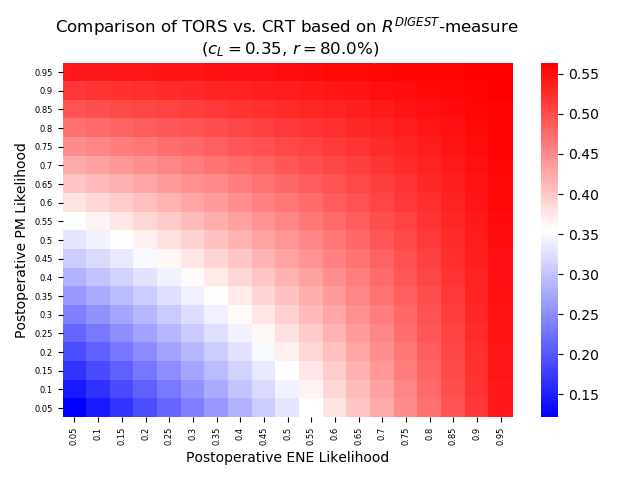
**

Figure B7: Expected deterioration in swallowing function due to TORS and definitive CRT based on long-term measures (left) MDADI, (center) MDASI, (right) DIGEST. $\Delta_{S}^{MDADI}$: MDADI-based absolute short-term deterioration; $\Delta_{S}^{MDASI}$: MDASI-based absolute short-term deterioration; $D^{DIGEST}$: DIGEST-based absolute short-term deterioration in swallowing function; $c_{S}$: cut-off value for TORS; and $r:$ risk associated with TORS for $p_{TM}^{+}=0.1.$

- 1. **Long-term outcomes (**$\boldsymbol{p}_{\boldsymbol{TM}}^{\mathbf{+}}\mathbf{=0.9}$**)**

**
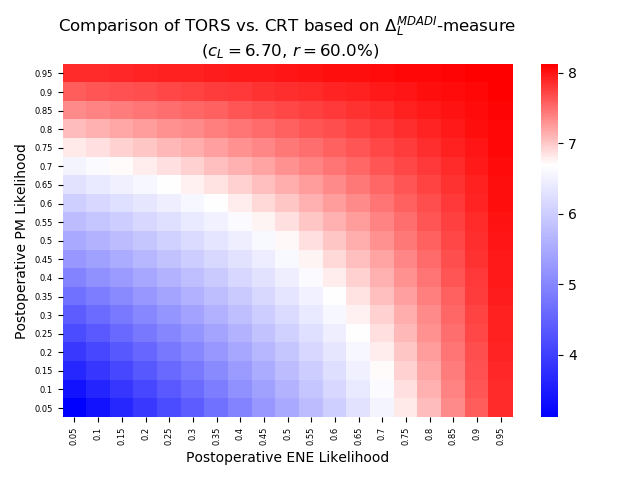

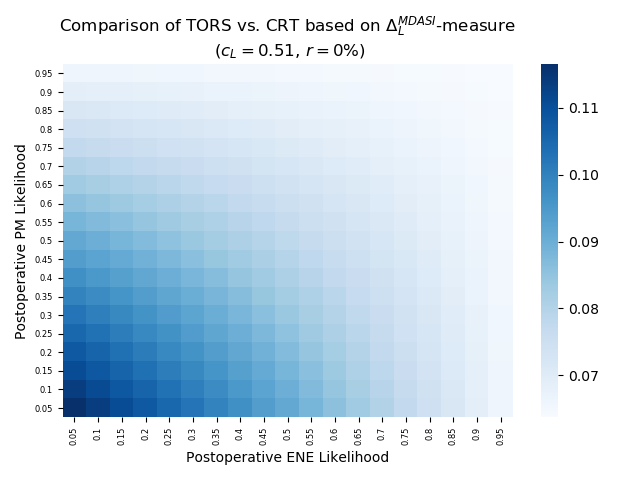

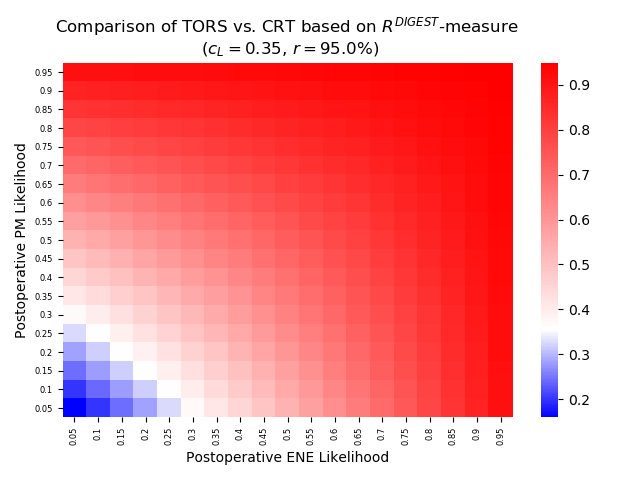
**

Figure B8: Expected deterioration in swallowing function due to TORS and definitive CRT based on long-term measures (left) MDADI, (center) MDASI, (right) DIGEST. $\Delta_{S}^{MDADI}$: MDADI-based absolute short-term deterioration; $\Delta_{S}^{MDASI}$: MDASI-based absolute short-term deterioration; $D^{DIGEST}$: DIGEST-based absolute short-term deterioration in swallowing function; $c_{S}$: cut-off value for TORS; and $r:$ risk associated with TORS for $p_{TM}^{+}=0.9.$

Table B2 summarizes the risk level variation associated with TORS (when compared to definitive CRT), as a function of tumor resection margin, in the absence of pre-therapy information about the postoperative ENE and PM likelihoods.

Table B2: Sensitivity of risk level associated with TORS (vs. definitive CRT) as a function of postoperative tumor resection margin. $p_{TM}^{+}$: probability of having tumor resection margin > 2mm; $r$: TORS risk level; (I): Insensitive to $p_{TM}^{+}$; (HS): Highly sensitive to $p_{TM}^{+}$; (RS) Relatively sensitive to $p_{TM}^{+}$; (LS): Low sensitive to $p_{TM}^{+}$;

|  | $\boldsymbol{p}_{\boldsymbol{TM}}^{\mathbf{+}}$ **= 50%** | **Risk sensitivity to** $\boldsymbol{p}_{\boldsymbol{TM}}^{\mathbf{+}}$ | |  |
| --- | --- | --- | --- | --- |
| **Short-term toxicity level** | $0\%\leq r\leq45\%$  TORS carries average risk  (almost conclusive across all instruments) | MDADI | $r=0\%$ (I) | |
|  |  | MDASI | $r=0\%$ (I) | |
|  |  | DIGEST | $0\%\leq r\leq76\%$ (LS) | |
| **Long-term toxicity level** | $0\%\leq r\leq91\%$  TORS risk varies based on the instrument  (inconclusive across all instruments) | MDADI | $0\%\leq r\leq60\%$ (HS) | |
|  |  | MDASI | $r=0\%$ (I) | |
|  |  | DIGEST | $80\%\leq r\leq95\%$ (LS) | |
