# Supplementary material for: Optimized decision support for selection of transoral robotic surgery or (chemo)radiation therapy based on posttreatment swallowing toxicity
Source: Cancer Med. 2022 Oct 13;12(4):5088–98. doi: 10.1002/cam4.5253 (PMC9972156; doi:10.1002/cam4.5253)
Supplement: Supplementary file 3 — Appendix C [file CAM4-12-5088-s002.docx]

**Appendix C: DIGEST baseline grades evolution within 3-6 and 18-24 months**

Table C1: The evolution of DIGEST baseline grades within 3-6 moths and within 18-24 months post therapy.

| RT | | Within 3-6 months | | | | |  | RT | | Within 18-24 months | | | | |
| --- | --- | --- | --- | --- | --- | --- | --- | --- | --- | --- | --- | --- | --- | --- |
|  |  | 0 | 1 | 2 | 3 | 4 |  |  |  | 0 | 1 | 2 | 3 | 4 |
| Baseline | 0 | 8 | 6 | 1 |  |  |  | Baseline | 0 | 3 | 2 | 1 |  |  |
|  | 1 | 3 | 2 | 3 |  |  |  |  | 1 | 3 | 2 |  |  |  |
|  | 2 |  |  |  |  |  |  |  | 2 |  |  |  |  |  |
|  | 3 |  |  |  |  |  |  |  | 3 |  |  |  |  |  |
|  | 4 |  |  |  |  |  |  |  | 4 |  |  |  |  |  |
| (a) | | | | | | |  | (b) | | | | | | |
|  |  |  |  |  |  |  |  |  |  |  |  |  |  |  |
| CRT | | Within 3-6 months | | | | |  | CRT | | Within 18-24 months | | | | |
|  |  | 0 | 1 | 2 | 3 | 4 |  |  |  | 0 | 1 | 2 | 3 | 4 |
| Baseline | 0 | 50 | 35 | 14 | 4 |  |  | Baseline | 0 | 34 | 15 | 4 | 1 |  |
|  | 1 | 7 | 3 | 5 | 1 |  |  |  | 1 | 3 | 6 | 2 | 1 |  |
|  | 2 | 14 | 5 |  |  |  |  |  | 2 |  |  |  |  |  |
|  | 3 |  |  |  | 1 |  |  |  | 3 |  |  |  |  |  |
|  | 4 |  |  |  |  |  |  |  | 4 |  |  |  |  |  |
| (c) | | | | | | |  | (d) | | | | | | |
|  |  |  |  |  |  |  |  |  |  |  |  |  |  |  |
| TORS+RT | | Within 3-6 months | | | | |  | TORS+RT | | Within 18-24 months | | | | |
|  |  | 0 | 1 | 2 | 3 | 4 |  |  |  | 0 | 1 | 2 | 3 | 4 |
| Baseline | 0 | 3 | 8 | 2 |  |  |  | Baseline | 0 |  | 3 | 3 |  |  |
|  | 1 |  | 2 |  | 1 |  |  |  | 1 |  |  | 1 |  |  |
|  | 2 |  |  |  |  |  |  |  | 2 |  |  |  |  |  |
|  | 3 |  |  |  |  |  |  |  | 3 |  |  |  |  |  |
|  | 4 |  |  |  |  |  |  |  | 4 |  |  |  |  |  |
| (e) | | | | | | |  | (f) | | | | | | |
|  |  |  |  |  |  |  |  |  |  |  |  |  |  |  |
| TORS+CRT | | Within 3-6 months | | | | |  | TORS+CRT | | Within 18-24 months | | | | |
|  |  | 0 | 1 | 2 | 3 | 4 |  |  |  | 0 | 1 | 2 | 3 | 4 |
| Baseline | 0 | 4 | 3 | 1 |  |  |  | Baseline | 0 | 2 | 2 | 1 |  |  |
|  | 1 | 1 |  |  |  |  |  |  | 1 |  | 1 |  |  |  |
|  | 2 |  |  | 1 |  |  |  |  | 2 |  |  |  |  |  |
|  | 3 |  |  |  |  |  |  |  | 3 |  |  |  |  |  |
|  | 4 |  |  |  |  |  |  |  | 4 |  |  |  |  |  |
| (g) | | | | | | |  | (h) | | | | | | |
|  |  |  |  |  |  |  |  |  |  |  |  |  |  |  |
| TORS | | Within 3-6 months | | | | |  | TORS | | Within 18-24 months | | | | |
|  |  | 0 | 1 | 2 | 3 | 4 |  |  |  | 0 | 1 | 2 | 3 | 4 |
| Baseline | 0 | 11 | 5 |  |  |  |  | Baseline | 0 | 8 | 1 |  |  |  |
|  | 1 | 3 | 4 |  |  |  |  |  | 1 | 2 | 1 |  |  |  |
|  | 2 | 1 |  |  |  |  |  |  | 2 |  |  |  |  |  |
|  | 3 |  |  |  |  |  |  |  | 3 |  |  |  |  |  |
|  | 4 |  |  |  |  |  |  |  | 4 |  |  |  |  |  |
| (i) | | | | | | |  | (j) | | | | | | |
